# *Brucella neotomae* Infection in Humans, Costa Rica 

**DOI:** 10.3201/eid2306.162018

**Published:** 2017-06

**Authors:** Marcela Suárez-Esquivel, Nazareth Ruiz-Villalobos, César Jiménez-Rojas, Elías Barquero-Calvo, Carlos Chacón-Díaz, Eunice Víquez-Ruiz, Norman Rojas-Campos, Kate S. Baker, Gerardo Oviedo-Sánchez, Ernesto Amuy, Esteban Chaves-Olarte, Nicholas R. Thomson, Edgardo Moreno, Caterina Guzmán-Verri

**Affiliations:** Universidad Nacional, Heredia, Costa Rica (M. Suárez-Esquivel, N. Ruiz-Villalobos, C. Jiménez-Rojas, E. Barquero-Calvo, E. Víquez-Ruiz, E. Moreno, C. Guzmán-Verri);; Universidad de Costa Rica, San José, Costa Rica (E. Barquero-Calvo, C. Chacón-Díaz, N. Rojas-Campos, G. Oviedo-Sánchez, E. Chaves-Olarte, E. Moreno, C. Guzmán-Verri);; Wellcome Trust Sanger Institute, Hinxton, UK (K.S. Baker, N.R. Thomson);; Caja Costarricense del Seguro Social, Puntarenas, Costa Rica (E. Amuy)

**Keywords:** *Brucella*, *Brucella neotomae*, zoonoses, neurobrucellosis, bacteria, Costa Rica, brucellosis, neurological infections

## Abstract

Several species of *Brucella* are known to be zoonotic, but *B. neotomae* infection has been thought to be limited to wood rats. In 2008 and 2011, however, *B. neotomae* was isolated from cerebrospinal fluid of 2 men with neurobrucellosis. The nonzoonotic status of *B. neotomae* should be reassessed.

Members of the genus *Brucella* are the infectious agents of brucellosis, a neglected disease responsible for economic losses resulting from abortion and low performance in production animals (*1*). The 4 species mainly responsible for this widespread bacterial zoonosis are *B. melitensis*, *B. abortus*, *B. suis*, and to a lesser extent *B. canis*. Underdiagnosis and limited awareness of the disease among healthcare practitioners is common in many countries (*1*).

*B. neotomae*, isolated in 1957 from wood rats (*Neotoma lepida*) in North America (*2*), has been considered nonzoonotic (*3*). It has been isolated from target organs of experimentally infected mice and guinea pigs (*4*,*5*). We report the isolation of *B. neotomae* from cerebrospinal fluid samples from 2 human patients with neurobrucellosis.

## The Study

In 2008, a *Brucella* sp. isolate was submitted to the Tropical Diseases Research Center at the Universidad de Costa Rica. This isolate (denoted strain bneohCR2) was cultured from a cerebrospinal fluid sample obtained from a 64-year-old male patient at one of the main hospitals in San José, Costa Rica. In 2011, another isolate (denoted strain bneohCR1) was recovered from a cerebrospinal fluid sample from a 51-year-old male patient at a regional hospital in Costa Rica. As is common for other patients with brucellosis, the blood leukocyte count for each patient was almost within the reference range, and C-reactive protein level was within reference range. Both patients showed clinical signs compatible with neurobrucellosis (*6*), had positive Rose Bengal test results, and recovered after receiving 1 month of streptomycin (750 mg/d) and 3 months of doxycycline (100 mg/12 h).

**Figure 1:**
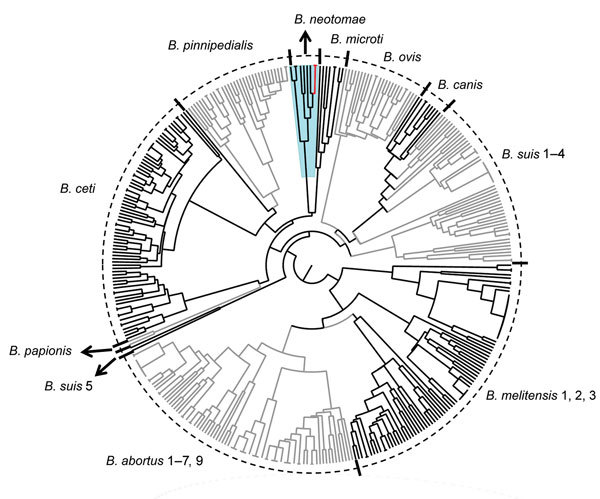
Dendrogram based on multiple locus variable number of tandem repeats–16-loci panel analysis of *Brucella* spp. (http://mlva.u-psud.fr/brucella/) and clinical isolates from human cerebrospinal fluid samples from 2 patients with brucellosis. The isolates bneohCR1 and bneohCR2 (red branches) showed a pattern consistent with previously reported profiles for *Brucella neotomae* (blue shading). Black, gray, and tic marks are used to differentiate between adjacent species. Arrows separate small neighboring clusters and indicate the *B. neotomae* cluster.

Further bacteriologic analysis (*7*,*8*) confirmed that the isolates were a *Brucella* sp. (Table). Real-time PCR high-resolution melting analysis (*9*) confirmed genus designation but was inconclusive regarding species designation. Bruce-ladder multiplex PCR (*10*) and multiple loci variable number of tandem repeats–16-loci panel analysis (http://mlva.u-psud.fr/brucella/; Figure 1) indicated that the profiles for both DNA isolates corresponded to profiles for *B. neotomae*. Analysis of bneohCR2 by multiplex single-nucleotide polymorphism (SNP) primer extension assay (*11*) and by matrix-assisted laser desorption/ionization time-of-flight mass spectrometry of protein extracts (*12*) (Technical Appendix Table 1) confirmed that the isolate was *B. neotomae*.

**Table:** Differential biochemical profile of *Brucella* isolates from 2 men with neurobrucellosis, Costa Rica, 2008 and 2011

Analysis	bneohCR1	bneohCR2
Biochemical tests		
Oxidase	-	–
Citrate utilization	–	–
Nitrate reduction	+	+
CO_2_ required	–	–
H_2_S production	+	+
Urease activity, h	0–0.5+	0–0.5+
Growth in presence of dyes		
Thionin 20 μg/mL	–	–
Basic fuchsin 20 μg/mL	–	–
A	+	+
M	–	–

**Figure 2:**
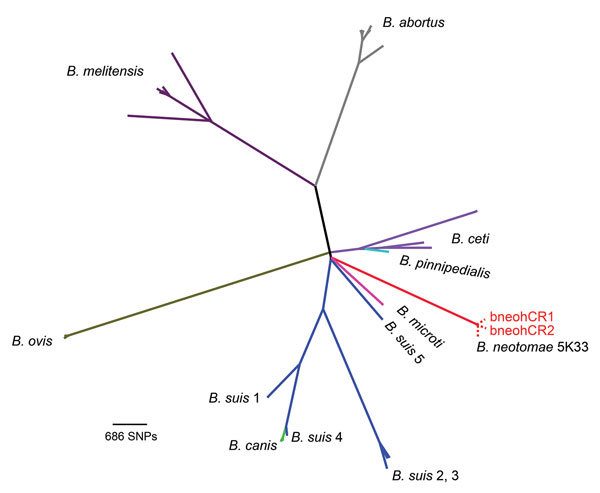
Phylogenetic tree based on 34,307 single-nucleotide polymorphisms (SNPs) found among 51 *Brucella* genome sequences. The clinical isolates bneohCR1 and bneohCR2 cluster with *B. neotomae* 5K33 and differ by 164 SNPs. A different color is used to represent each *Brucella* species. Dotted red lines denote the 3 *B. neotomae* isolates, which overlap at the tip of the branch because of the high identity among them.

We performed whole-genome sequencing of bneohCR1 and bneohCR2 and resequencing of reference strain *B. neotomae* 5K33. Sequencing data were deposited at the European Nucleotide Archive (http://www.ebi.ac.uk/ena/) under accession codes ERS1563929 (bneohCR1), ERS1563928 (bneohCR2), and ERS162447 (5K33). To place the bneohCR1 and bneohCR2 in a phylogenetic context, publicly available reads from 51 *Brucella* whole-genome sequences (Technical Appendix Table 2) were aligned and then mapped to *B. suis* 1330 by using SMALT version 0.5.8 (ftp://ftp.sanger.ac.uk/pub/resources/software/smalt/). Reads from bneohCR1 and bneohCR2 genomes mapped to 98.6% of the *B. suis* 1330 genome. SNPs were called from the alignment by use of Samtools (http://samtools.sourceforge.net/), and 34,307 variable sites across all isolates were extracted by using SNP sites (*13*). The resulting alignment of SNPs was used for maximum-likelihood phylogenetic reconstruction by use of RAxML version 7.0.4 (https://github.com/stamatak/standard-RAxML). The generated phylogenetic tree confirmed that the bneohCR isolates clustered together with *B. neotomae* reference strain 5K33 (ENA accession no. JMSC01, assembly accession no. GCA_00742255.1) (Figure 2). Isolates bneohCR1 and bneohCR2 differed from the reference genome by 174 and 160 SNPs, respectively. This number of SNPs is smaller than that between *B. abortus* 9–941 and *B. abortus* 2308 (214 SNPs), which are 2 well-recognized strains of the same *Brucella* species.

Analysis of 23 previously reported genomic islands or anomalous genomic loci (*14*) was performed for both bneohCR genomes. For this analysis, a “genomic-island pseudo-molecule” was constructed by concatenation of 23 genomic regions obtained from different *Brucella* genomes. BLAST (https://github.com/sanger-pathogens/Farm_blast) comparison of this pseudo-molecule and the bneohCR draft genomes, generated by assembly with Velvet (*15*), showed that the genomic loci known as 26.5 kb, 12 kb, and GI-6 that are absent in *B. neotomae* (*14*) are also absent in the queried genomes.

## Conclusions

This report of *B. neotomae* as a cause of zoonotic disease raises questions about possible underrepresentation of reported cases. This study also has implications for brucellosis diagnosis. Specifically, the differences among *B. neotomae* and the other *Brucella* species at the biochemical level are subtle. The major difference between *B. neotomae* and *B. abortus*, the main cause of human brucellosis in Costa Rica, is the presence of oxidase activity in *B. abortus*, which is assessed subjectively (*7*,*8*). Because *B. neotomae* has not, until now, been considered zoonotic, some cases of brucellosis reported as being caused by atypical zoonotic classical *Brucella* might have been misdiagnosed cases of *B. neotomae* infection. The introduction of whole-genome sequencing into the clinical field will thus improve diagnosis. 

A lack of epidemiologic information with regard to the 2 patients reported here precluded the investigation of exposure or contact with hosts known to harbor *B. neotomae*. Further studies are needed to establish which animals may act as reservoirs for *B. neotomae* in Costa Rica.

In summary, *B. neotomae* should be considered a zoonosis risk for infection in humans. Incorporation of molecular techniques for diagnosis will help resolve the *Brucella* genus homogeneity obtained when only biochemical assays are used.

Technical Appendix Protein mass values of *Brucella* reference strains and bneohCR2, and general information and accession numbers of the genomes included in the phylogenetic analysis.
