# Correction: Vol. 23, No. 6

**DOI:** 10.3201/eid2308.C22308

**Published:** 2017-08

**Keywords:** errata, erratum, correction

A subhead was missing and layout was incorrect in the Table for *Brucella neotomae* Infection in Humans, Costa Rica (M. Suárez-Esquivel et al.). The article has been corrected online (https://wwwnc.cdc.gov/eid/article/23/6/16-2018_article).

