# Supplementary material for: Brucella neotomae Infection in Humans, Costa Rica
Source: Emerg Infect Dis. 2017 Jun;23(6):997–1000. doi: 10.3201/eid2306.162018 (PMC5443450; doi:10.3201/eid2306.162018)
Supplement: Technical Appendix — Protein mass values of Brucella reference strains and bneohCR2, and general information and accession numbers of the genomes included in the phylogenetic analysis. [file 16-2018-Techapp-s1.pdf]

Article DOI: http://dx.doi.org/10.3201/eid2306.162018

Brucella neotomae Infection in Humans

Technical Appendix

Technical Appendix Table 1. Protein mass values of *Brucella* reference strains and bneohCR2*

Peak N°	Mass	Bce	Bpi	Bme	Bab	Bsu	Bca	Bov	Bmi	BneK	BneCR
1	2771	0	1	0	0	0	0	0	0	0	0
2	2919	0	0	0	0	1	0	0	0	0	0
3	3026	0	1	1	1	1	1	1	0	0	0
4	3181	1	1	1	0	1	1	0	0	0	0
5	3407	0	0	0	1	0	0	1	0	0	0
6	3700	1	1	1	1	1	1	1	1	1	1
7	3831	0	0	0	0	0	1	1	0	0	0
8	4126	0	0	0	1	0	0	1	0	0	0
9	4541	0	1	1	1	0	1	1	0	1	1
10	4558	1	0	0	1	1	0	0	1	0	0
11	4854	1	1	1	1	1	1	1	1	1	1
12	4898	0	0	1	0	1	1	0	0	0	0
13	5112	0	0	0	1	0	0	1	0	0	0
14	5135	1	1	1	1	1	1	0	0	1	1
15	5169	1	0	1	0	1	1	1	1	1	0
16	5546	1	1	1	0	0	0	0	0	0	0
17	5686	1	1	1	0	1	1	1	1	1	1
18	5772	1	1	0	0	0	1	0	0	0	0
19	5834	0	0	0	1	1	1	0	0	0	0
20	5876	1	1	0	1	0	0	1	0	1	1
21	5940	0	0	0	1	0	0	0	1	1	0
22	6039	0	0	1	0	1	0	0	1	0	0
23	6049	0	0	0	1	0	0	0	0	1	1
24	6150	0	0	0	1	1	0	0	0	0	0
25	6289	1	1	1	1	1	1	1	1	1	1
26	6322	0	0	1	0	1	0	0	0	0	0
27	6369	1	1	0	1	0	1	0	0	0	0
28	6420	0	0	1	1	1	0	0	0	0	0
29	6542	1	1	1	1	1	1	0	1	1	0
30	6676	1	1	1	1	1	1	1	0	1	1
31	7056	1	1	1	0	1	1	1	1	1	1
32	7183	1	1	1	1	1	0	0	0	1	0
33	7270	0	0	0	1	0	0	0	0	0	0
34	7309	0	0	0	0	0	1	0	0	0	0
35	7362	0	0	0	0	0	0	0	1	0	0
36	7400	1	1	1	0	1	1	1	1	1	1
37	7518	1	1	1	0	1	1	1	1	1	1
38	7660	1	0	1	0	1	1	1	0	1	1
39	7785	1	0	1	0	1	1	1	0	0	0
40	8045	1	1	1	0	1	1	1	1	0	0
41	8121	0	1	0	0	0	0	0	0	0	0
42	8192	1	0	1	1	1	1	1	0	1	0
43	8247	1	1	1	0	1	0	1	0	1	1
44	8589	0	0	0	0	1	0	0	0	0	0
45	8685	1	1	1	1	1	1	1	0	1	1
46	8730	1	1	1	1	1	1	0	1	1	1
47	8823	1	1	1	0	1	1	1	1	0	0
48	9082	0	1	1	0	0	1	1	0	1	1
49	9113	1	0	0	1	1	0	0	1	0	0
50	9319	1	1	1	1	1	1	1	1	1	0
51	9800	0	0	1	0	1	1	1	0	1	1
52	9827	0	0	0	1	0	0	0	1	0	0
53	9970	0	0	1	0	0	0	1	0	0	0
54	10085	1	1	0	0	1	1	0	1	1	1
55	10227	0	0	0	1	0	0	1	0	1	1
56	10271	1	1	1	1	1	1	0	1	0	0
57	10417	1	1	1	1	1	1	1	1	1	0

Peak N°	Mass	Bce	Bpi	Bme	Bab	Bsu	Bca	Bov	Bmi	BneK	BneCR

*Bce, *B. ceti* bmarCR17; Bpi, *B. pinnipedialis* B2/94; Bme, *B. melitensis* Rev1; Bab, *B. abortus* 2308; Bsu, *B. suis* s2; Bca, *B. canis* CR12; Bov, *B. ovis* PA; Bmi, *B. microti* CCM4915; BneK, *B. neotomae* 5K33, BneCR, *B. neotomae* bneohCR2; 0, absence; 1, presence.

Technical Appendix Table 2. General information and accession numbers of the genomes included in the phylogenetic analysis*

Strain ID	Sample	Host	Country	Accession number
B. abortus 9–941	ND	Bovine	USA	NC_006932.1 & NC_006933.1
B. abortus 2308	Aborted fetus	Bovine	USA	NC_007618.1 & NC_007624.1
B. abortus 2308W	Aborted fetus	Bovine	USA	ERS668782
B. abortus 01–0065	ND	Bison	USA	GCA_000413795.1
B. abortus 104M	Placenta	Cattle	China	GCA_001296965.1
B. abortus 134	Blood	Human	China	GCA_000298635.1
B. abortus CNBG 1432	Blood	Human	Argentina	GCA_000366525.1
B. abortus CNBG 308	Blood	Human	Argentina	GCA_000366545.1
B. abortus 3196	ND	ND	ND	GCA_000740945.1
B. abortus 63/144	ND	Human	France	GCA_000370025.1
B. abortus 63/59	ND	ND	Poland	GCA_000366605.1
B. abortus 63/75	ND	ND	ND	GCA_000740295.1
B. abortus 64/108	ND	Goat	India	GCA_000370085.1
B. canis ATCC 23365	Allantoic fluid of aborted puppy	Dog	ND	NC_010103.1 & NC_010104.1
B. canis HSK A52141	Blood	Dog	South Korea	GCA_000238195.1
B. canis Oliveri	ND	ND	Colombia	GCA_000530495.1
B. canis RM6/66	ND	ND	ND	GCA_000740335.1
B. canis SVA13	Aborted material	Dog	Sweden	GCA_000691585.1
Brucella ceti TE10759–12	Brain and spleen	Striped dolphin	Italy	CP006896 & CP006897
B. ceti TE28753	Brain	Striped dolphin	Italy	CP006898.1 & CP006899.1
B. ceti F23–97	ND	Bottle nose dolphin	France	NZ_AQKR00000000.1
B. melitensis 16M ATCC 23456	ND	Goat	USA	NC_003317.1 &NC_003318.1
B. melitensis ATCC 23457	ND	Goat	Turkey	NC_012441.1 &:NC_012442.1
B. melitensis M28	ND	Sheep	Vaccine in China	NC_017244.1 & NC_017245.1
B. melitensis M5–90	Vaccine	Vaccine from M28	China	NC_017246.1&NC_017247.1
B. melitensis NI	Aborted fetus	Bovine	China	NC_017248.1 & NC_017283.1
B. melitensis bv. 3 str. Ether	ND	ND	ND	NZ_CP007760.1 & NZ_CP007761.1
B. melitensis 20236	ND	ND	ND	NZ_CP008750.1 & NZ_CP008751.1
B. melitensis 2008724259	ND	ND	ND	NZ_CP016983.1 & NZ_CP016984.1
B. melitensis bv. 2 str. 63/9	ND	ND	ND	NZ_CP007789.1 & NZ_CP007788.1
B. microti CCM 4915	Systemic infection	Common voles	Czech Republic	NC_013119.1 & NC_013118.1
B. neotomae 5K33	Pooled tissue: lung, spleen, liver and kidney	Desert wood rat	Utah, USA	JMSC01 GCA_000742255.1 ERS1624467†
bneohCR1	CSF	Human	Costa Rica	ERS1563929†
bneohCR2	CSF	Human	Costa Rica	ERS1563928†
B. ovis ATCC 25840	Tissue	ND	Australia	NC_009505.1 & NC_009504.1
B. ovis IntaBari-2008–114–542	ND	ND	Argentina	GCA_000365985.1
B. ovis IntaBari-1993–758	ND	ND	Argentina	GCA_000366005.1
B. ovis IntaBari-2009–88–4	ND	ND	Argentina	GCA_000366045.1
B. ovis F8/05B	ND	ND	ND	GCA_000367085.1
Brucella pinnipedialis B2/94	Spleen	Common seal	Scotland	NC_015857.1 & NC_015858.1
B. suis 1330	ND	Swine	ND	NC_004310.3 & NC_004311.2
B. suis ATCC 23445	ND	Swine	Denmark	NC_010169.1 & NC_010167.1

Strain ID	Sample	Host	Country	Accession number
B. suis VBI22	Milk	Bovine	USA	NC_016797.1 & NC_016775.1
B. suis bv.1 str. S2	Vaccine	Vaccine in China	China	NZ_CP006961.1 & NZ_CP006962.1
B. suis bv. 2 PT09143	ND	Wild boar	Spain/Portugal	NZ_CP007691.1 &NZ_CP007692.1
B.*suis* bv. 3 str. 686	ND	ND	ND	NZ_CP007719.1 & NZ_CP007718.1
B. suis bv. 5 str. 513	ND	ND	ND	NZ_DS999724.1
B. suis bv. 4 str. 40	ND	ND	ND	NZ_GG703793.1
B. suis F5/05–4	ND	ND	ND	NZ_KB850877.1
Brucella sp. F5–99	Aborted fetus	Bottle nose dolphin	USA	NZ_ACFF00000000

*CSF, cerebrospinal fluid; ND, no data available.
†Isolates and/or WGS described in this study.
